# Summary of papers presented at the 2012 seventh international cough symposium

**DOI:** 10.1186/1745-9974-9-13

**Published:** 2013-05-02

**Authors:** Peter V Dicpinigaitis, Giovanni A Fontana, Lu-Yuan Lee, Milos Tatar

**Affiliations:** 1 Albert Einstein College of Medicine and Montefiore Medical Center, Bronx, NY, USA; 2 Department of Internal Medicine, University of Florence, Florence, Italy; 3 Department of Physiology, University of Kentucky Medical Center, 800 Rose Street, Lexington, KY 40536-0298, USA; 4 Department of Pathophysiology, Jessenius Faculty of Medicine, Comenius University, Martin, Slovakia

**Keywords:** Cough suppressant, Stem cells, Mouse, Brainstem, High altitude, GERD, Aspiration pneumonia, Symptom assessment, Treatment

## Abstract

Twenty six papers were presented as posters in the Seventh International Symposium on Cough; 12 papers were presented in the Basic Science of Cough session, and 14 papers presented in the Clinical Science of Cough session. These papers explored a wide spectrum of cough-related areas including pathophysiological mechanisms, treatment and detection of cough, and symptom assessment and perception, and were grouped into several general themes for facilitate the discussion. Studies presented in these posters have provided new information that should improve our knowledge on the basic physiology and pharmacology of cough, and the peripheral and central neural mechanisms involved in the generation of the cough motor pattern. In addition, in the clinical science section, studies reporting potential new anti-tussive agents and further characterisation of cough symptoms and perception have provided a base for the fruitful strategies for the development of novel anti-tussive therapies and cough management.

## Introduction

The Seventh International Symposium on Cough convened in London, UK, in July, 2012. As in previous meetings, investigators presented work in poster format, followed by an oral session devoted to discussion of those posters in the presence of the full Symposium audience. Twelve papers were presented in the Basic Science of Cough session, and 14 papers presented in the Clinical Science of Cough session; the 26 papers with their authors are listed in Table 1. These papers explored a wide spectrum of cough-related areas including mechanisms, treatment and detection of cough, and symptom assessment and perception, and were grouped into several general themes to facilitate the discussion.

**Table 1:** List of papers with authors

**A.**	**Basic Science of Cough:**
	**Chen L, Lomask J, Lai K, Jiang B, and Zhong N.** Detection of mouse cough based on sound monitoring.
	**Clarke R, Curtis TA, Cosby SL, McGarvey L, and Lundy FT.** An in vitro model for pain and neurogenic inflammation in the oro-facial region and upper airways.
	**Hanacek J, Tatar M, Stransky A, and Tomori Z.** Professor John Widdicombe - spiritus movens of Slovak experimental respirology.
	**Iwata T, Ito I, Niimi A, Ikegami K, Marumo S, Tanabe N, Nakaji H, Matsumoto H, Oguma T, Inoue H, Tajiri T, Nagasaki T, Kanemitsu Y, Kamei J, Setou M, and Mishima M.** Mice with immotile cilia spontaneously cough due to mechanical stimuli of postnasal drip.
	**Mutolo D, Bongianni F, Cinelli E, Giovannini MG, and Pantaleo T.** Activation of ERK1/2 in the caudal nucleus tractus solitarii is required for the mediation of cough reflex responses in the rabbit.
	**Noguchi T, Soeda F, Shirasaki T, Akaike N, and Takahama K.** Further studies on properties of antitussive activity of human coagulation factor (f-XIa) in guinea pigs.
	**Poliacek I, Simera M, Veternik M, Machac P, Barani H, Visnovcova N, and Jakus J.** Medullary midline raphe contributes to phase duration control of tracheobronchial coughing - codeine trials in cat.
	**Simera M, Poliacek I, Veternik M, Dobrolubov B, Barani H, Visnovcova N, and Jakus J.** Effect of kainic acid medullary raphe lesions on cough in anesthetized rabbits.
	**Takahama K, Noguchi T, Kamei S, lto Y, and Akaike N.** Discovery of a potent cough suppressant substance extracted from the plasma in humans.
	**Tomori Z, Donic V, and Jakus J.** Perspective applications of the aspiration, expiration and cough reflexes, as well as their voluntary counterparts.
	**Varechova S, Demoulin B, Inglebert Y, LeTuan T, Leblanc A-L, Schweitzer C, and Marchal F.** Long term sensitisation of cough and expiration reflex in adult rabbits by 48 hour postnatal normobaric hyperoxia.
	**Veterník M, Šimera M, Jakuš J, and Poliaček I.** Effect of moving average window width on integration of EMG signal.
**B.**	**Clinical Science of Cough:**
	**Buday, T, Brozmanova, M, Biringerova, Z, Gavliakova, S, Poliacek, I, Calkovsky, V, Shetthalli, MV, Plevkova, J:** Urge to cough, cough sensitivity and intensity after nasal TRPM8 and TRPA1 agonists challenges
	**Ebihara, S, Gui, P, Ebihara, T, Kashiwazaki, N, Ito, K, Kanezaki, M, Kohzuki, M:** Urge-to-cough and dyspnoea conceal perception of pain in healthy adults
	**Chellini, E, Gonfiotti, A, Magni, C, Innocenti, M, Jaus, M, Lavorini, F, De Francisci, A, Fontana, G, Macchiarini, P**: Membranous tracheobronchial reduction surgery for excessive dynamic airway collapse associated with intractable chronic cough
	**Ito, I, Ishida, T, Tachibana, H, Tomioka, H, Kadowaki, S, Tanabe, N, Niimi, A, Mishima, M:** Presence of dry cough symptom may prevent recurrent fever in patients treated for pneumonia
	**Kanemitsu, Y, Nimi, A, Matsumoto, H, Ito, I, Oguma, T, Inoue, H, Tajiri, T, Iwata, T, Nagasaki, T, Mishima M:** Cough assessed by the Leicester Cough Questionnaire (LCQ) in patients with cough variant asthma: impact of sex and gastric dysmotility
	**La-Crette, J, Lee, KK, Saito, J, Hull, J, Chung, KF, Birring, SS:** The development of a cough hypersensitivity questionnaire (CHQ)
	**Mason, NP:** Altitude-related cough
	**Ogawa, H, Fujimura, M, Takeuchi, Y, Makimura, K**: The significance of sensitization to *Bjerkandera adusta* in the successful treatment of chronic idiopathic cough patients
	**Ohkura, N, Fujimura M:** Relation between bronchoconstriction-triggered cough and protective reaction against bronchoconstriction
	**Ryan, NM, Birring, SS, Gibson, PG**: Gabapentin treatment for refractory chronic cough: a randomized controlled trial
	**Smith, JA, Murdoch, R, Newlands, A, Khalid, S, Smart, K, Kelsall, A, Holt, K, Dockry, R, Woodcock, A**: The impact of a selective oral TRPV1 antagonist in patients with chronic cough
	**Wei, ET, Vitins, P:** Design of topical sensory agonists for cough
	**Xu, X, Chen, Q, Liang, S, Lv, H, Qiu, Z:** Prediction of gastroesophageal reflux-induced cough with questionnaire for patients with upper gastrointestinal symptoms
	**Zhang, Q, Li, N, Liu, M, Liao, L, He, M, Feng, M, Li, J:** The effect of temperature during sputum processing on inflammatory markers

## Basic science of cough papers chaired by Drs. Lu-Yuan Lee and Milos Tatar

### In honour of John Widdicombe

The Seventh Symposium on Cough was dedicated in honour of John Widdicombe, the leading scientist in basic cough research from early 1950s of last century to 2011. He was the only scientist from western countries who opened the window through the iron curtain during the cold war, and respiratory physiologists from Slovakia were able to visit and work in his laboratories. The poster presented by Slovak respirologists, Hanacek J, Tatar M, Stransky A and Tomori Z, expressed the gratitude to Professor Widdicombe for invitations of numerous Slovak respirologists to study in his laboratories in Oxford and London, and for his important and invaluable contributions to establishing the Slovak Experimental Respirology School (**Hanacek J, Tatar M, Stransky A, and Tomori Z.** Professor John Widdicombe - spiritus movens of Slovak experimental respirology). Respiratory researchers in Slovakia were profoundly influenced by the stimulating ideas, visions and personality of Professor Widdicombe.

### Physiological and pharmacological studies of cough

The study by Takahama and co-workers demonstrated that a coagulation factor XIa (F-XIa) of human plasma could effectively suppress cough reflexes elicited by inhalation of citric acid aerosol and by mechanical stimulation to larynx and carina in lightly anesthetized guinea-pigs (**Takahama K, Noguchi T, Kamei S, lto Y, and Akaike N.** Discovery of a potent cough suppressant substance extracted from the plasma in humans). In an accompanying study from the same institute, Noguchi et al. further demonstrated a potent inhibitory effect of F-XIa on the cough response to citric acid aerosol in guinea pigs that developed sub-acute bronchitis after chronic exposure to sulfur dioxide (500 ppm, 2 hours each day for 7 days) (**Noguchi T, Soeda F, Shirasaki T, Akaike N, and Takahama K.** Further studies on properties of antitussive activity of human coagulation factor (f-XIa) in guinea pigs). Interestingly, F-XIa inhibited the codeine-resistant cough response to acid in guinea pigs treated with enalapril, an angiotensin-converting enzyme inhibitor. In contrast, it did not inhibit cough reflex triggered by electrical stimulation of the superior laryngeal nerve in normal guinea pigs, which could be suppressed by codeine. Based on these results, these investigators suggested that the anti-tussive action of F-XIa is on the peripheral site(s) of the cough reflex pathways. This finding is promising because the majority of the existing anti-tussive drugs with high cough-suppressant efficacy act primarily on the central components of cough reflex pathways and are known for generating various centrally-mediated side effects.

In the development of therapeutic agents for clinical use, the validity of data obtained from animal studies must be evaluated on the basis of its applicability and reproducibility in humans. In the last several decades, scientists have been searching for ideal animal models for studying the pathophysiological mechanisms involved in chronic cough and for testing and developing anti-tussive agents. Clarke and co-workers have presented an interesting study of the cells derived from human dental pulp stem cells that expresses neuronal marker PGP9.5, and transient receptor potential vanilloid type 1 (TRPV1) and TRPV4 channels (**Clarke R, Curtis TA, Cosby SL, McGarvey L, and Lundy FT.** An in vitro model for pain and neurogenic inflammation in the oro-facial region and upper airways). Their preliminary data demonstrated that these neuron-like cells can be utilized as “peripheral neuronal equivalents” for in-vitro study of human neurons. A further characterization of the electrophysiological and biochemical properties of these cells should reveal the potential and significance of this cell model for the future studies of airway sensory neurons (e.g., cough sensors).

### Animal models for study of cough

Questions remain as to whether mice can serve as a good animal model for studying cough, which is important and relevant because the various knockout mouse models currently available or that can be generated to target specific receptors and signaling pathways. The study by Chen and co-workers described a system to monitor and analyze the cough responses in unanesthetized and unrestrained mice based upon a computer analysis of the sound signals recorded (**Chen L, Lomask J, Lai K, Jiang B, and Zhong N.** Detection of mouse cough based on sound monitoring). Iwata and co-workers have previously reported that cough- or sneeze-like responses in tubulin tyrosine ligase-like family member 1 (Ttll1) gene knockout (KO) mice with airway ciliary motility disorders and the accompanying accumulation of mucus in the nasal cavity [1].

In this meeting, they reported additional data obtained from recent experiments using the whole-body plethysmography (**Iwata T, Ito I, Niimi A, Ikegami K, Marumo S, Tanabe N, Nakaji H, Matsumoto H, Oguma T, Inoue H, Tajiri T, Nagasaki T, Kanemitsu Y, Kamei J, Setou M, and Mishima M.** Mice with immotile cilia spontaneously cough due to mechanical stimuli of postnasal drip). The results of their study suggested that the spontaneous and persistent coughs observed in Ttll1-KO mice were related to the post-nasal drips that probably activated the pharyngeal and/or laryngeal rapidly adapting receptors in these mice. In the study of Varechova and co-workers, the cough sensitivity to mechanical stimulation of tracheal mucosa was significantly enhanced in the anesthetized rabbits after they were exposed to hyperoxia (O_2_ concentration >93%) for 48 hours in their early postnatal life (the first 8 days after birth) (**Varechova S, Demoulin B, Inglebert Y, LeTuan T, Leblanc A-L, Schweitzer C, and Marchal F.** Long term sensitisation of cough and expiration reflex in adult rabbits by 48 hour postnatal normobaric hyperoxia). Although the mechanism underlying this difference was not known, the authors suggested that the same pathogenic mechanism may be responsible for the chronic cough frequently observed in school children who were born prematurely and therefore exposed to hyperoxia in the postnatal stage.

### Interaction of cough and other respiratory reflexes

The potential of applications of the aspiration, expiration, and cough reflexes for clinical diagnosis and the]rapy of various respiratory disorders were presented by Tomori and co-workers (**Tomori Z, Donic V, and Jakus J.** Perspective applications of the aspiration, expiration and cough reflexes, as well as their voluntary counterparts). In cats, aspiration reflex can be provoked even in the premortal gasping state and capable of reversing transient agonal state, manifesting progressive atrio-ventricular blockade, severe bradycardia and hypotension caused by asphyxia. Furthermore, they study demonstrated that voluntary counterparts of these powerful reflexes could be used in prevention of these functional disorders. For example, the bronchospasm provoked by methacholine inhalation in asthmatics is significantly reduced by preceding voluntary sniffs. In addition, the increased cough sensitivity of asthmatic children is also inhibited by preceding voluntary sniffs.

### Central cough mechanisms

Three posters dealt with central nervous mechanisms regulating cough. A role for extracellular signal regulated kinases-1 and −2 (ERK1/2) pathway in central processing of tussive inputs was reported in the study by Mutolo and co-workers by microinjections of an inhibitor (U0126) into the caudal nucleus tractus solitarii (cNTS) on pentobarbitone anesthetized, spontaneously breathing rabbits (**Mutolo D, Bongianni F, Cinelli E, Giovannini MG, and Pantaleo T.** Activation of ERK1/2 in the caudal nucleus tractus solitarii is required for the mediation of cough reflex responses in the rabbit). Marked reduction or complete ablation (concentration dependent response) of the cough response induced by both mechanical and chemical stimulations of the tracheobronchial tree was assessed. This procedure did not affect either the Breuer-Hering inflation reflex, the pulmonary chemoreflex or the sneeze reflex. This study represents a first step toward more comprehensive investigations on the MAPK involvement in the transduction of cough-related extracellular stimuli into intracellular posttranslational and transcriptional responses.

The contribution of medulary midline raphe in regulation of cough was presented by two posters. The study by Poliacek and co-workers decribes that microinjections of codeine in the medullary raphe reduced amplitudes of abdominal muscle EMG and the cough expiratory phase duration, whereas the cough number, amplitudes of esophageal pressure during cough, inspiratory and other temporal cough parameters and cardiorespiratory characteristics were not altered significantly (**Poliacek I, Simera M, Veternik M, Machac P, Barani H, Visnovcova N, and Jakus J.** Medullary midline raphe contributes to phase duration control of tracheobronchial coughing - codeine trials in cat). However, intravenous injections of codeine reduced the peak expiratory esophageal pressure during coughing by approximately 50% and the cough number and amplitudes of abdominal EMG moving averages during coughing to approximately 30% of control values. The same group of investigators also recorded parameters of mechanically-induced cough, expiratory reflex and sneezing after the microinjections of kainic acid in the medullary raphe midline in anesthetized spontaneously breathing rabbits (**Simera M, Poliacek I, Veternik M, Dobrolubov B, Barani H, Visnovcova N, and Jakus J.** Effect of kainic acid medullary raphe lesions on cough in anesthetized rabbits). Kainic acid reduced the number of cough and the intensity of expiratory strength of cough, expiratory reflex and sneezing. Their results suggest complex and diverse role of neurons located in the medullary raphé in control of expression and motor pattern generation of cough and motor responses of other respiratory defense reflexes (e.g., sneeze, expiration reflex).

The effect of moving average window width on the range of values of rectified and integrated EMG signals was tested on a theoretical model presented by Veternik and co-workers (**Veterník M, Šimera M, Jakuš J, and Poliaček I.** Effect of moving average window width on integration of EMG signal). Their results were consistent with experiences obtained from analyses of experimental data in animals. This approach is also used to test the accuracy of methods for data analysis and parameters obtained on the variability of moving average data depending on the number of spikes integrated and the width of moving average window. Modeling of cough generating neuronal network and computer simulations of motor output during reflex behaviors provide deeper insight of the topic, offer an easier way to test hypotheses about the function of neuronal circuits and an important feedback for experimental studies.

## Clinical science of cough papers chaired by Drs. Peter Dicpinigaitis and Giovanni Fontana

### Mechanisms

A mechanism that remains poorly understood is that of altitude-related cough. A widely held belief had been that cough induced by ascent to high altitudes is caused by inspiration of cold, dry air [2]. This concept was refuted in an experiment by Mason (**Mason, NP**: Altitude-related cough), in which subjects were studied in a hypobaric chamber mimicking an ascent to Mt. Everest (8848 m). Despite temperature and humidity being carefully controlled, at 8000 m conditions subjects demonstrated increase in nocturnal cough frequency and enhancement of cough reflex sensitivity to inhaled citric acid. Possible explanations for these observations include subclinical pulmonary edema and drying of the respiratory mucosa due to water loss.

The relationship between cough and bronchoconstriction has been an area of considerable scientific inquiry for decades [3]. Ohkura and Fujimura hypothesized that bronchoconstriction-triggered cough would be associated with a protective effect against bronchoconstriction (**Ohkura, N, Fujimura M**: Relation between bronchoconstriction-triggered cough and protective reaction against bronchoconstriction). The investigators evaluated bronchial responsiveness and cough induction by inhaled methacholine in healthy volunteers. Measured recovery rates of PEF40 and FEV1 after methacholine challenge demonstrated that a higher induced cough number protected against mild, though not more severe, bronchoconstriction.

Insight into neural mechanisms of cough can serve as a starting point for the conception and development of potential antitussive drugs. Wei and Vitins (**Wei, ET, Vitins, P:** Design of topical sensory agonists for cough) studied 9^th^ (glossopharyngeal) and 10^th^ (vagal) cranial nerve afferents from the upper oropharynx. The authors speculate that a molecule designed as an agonist with a vigorous sensory impact on the oropharyngeal surface has the potential to suppress cough by a number of mechanisms, including: evoking pharyngeal swallowing reflexes that are incompatible with cough; evoking sensations that override tickling/itch in the throat; and, creating gating signals in the brainstem, where the 9^th^ and 10^th^ nerve afferents converge. Using their particular design and screening strategy, the investigators have identified multiple candidate molecules as potential cough suppressants.

Zhang and colleagues offered methodological insights into the measurement of cellular and biochemical inflammatory markers that may be relevant to cough induction (**Zhang, Q, Li, N, Liu, M, Liao, L, He, M, Feng, M, Li, J**: The effect of temperature during sputum processing on inflammatory markers). It has not been established whether the temperature at which induced sputum is processed is significant in terms of mediator detection and differential cell counts. Evaluating specimens from 8 asthmatic subjects, the investigators demonstrated that sputum processing at 37°C significantly reduced detectable concentrations of eosinophil cationic protein (ECP) and myeloperoxidase (MPO), thus prompting the recommendation that sputum be processed at 4°C to more accurately reflect levels of inflammatory markers.

### Symptom assessment and perception

One missing tile in the puzzle of cough assessment may be a reliable methodology to unmask pathological cough reflex sensitivity. Currently available methods, including the capsaicin cough challenge that is widely used for cough assessment, have proved to be easy to perform, well tolerated and reproducible; however, the methods have failed to discriminate between health and disease [4]. An interesting clinical study by La-Crette and Colleagues (**La-Crette, J, Lee, KK, Saito, J, Hull, J, Chung, KF, Birring, SS**: The development of a cough hypersensitivity questionnaire (CHQ) aimed at establishing the prevalence and severity of laryngeal sensations and cough triggers in patients with chronic cough of unexplained origin, in patients with cough associated with chronic respiratory diseases, and in healthy controls. The investigation was based on a newly developed questionnaire, the CHQ, for the assessment of prevalence and severity of common cough triggers and respiratory sensations. Patients and controls underwent assessments of their cough-related quality of life by means of the Leicester Cough Questionnaire (LCQ), a conventional capsaicin challenge with recordings of the cough threshold and intensity of the urge to cough (VAS score), as well as administration of the CHQ. The new and interesting finding was that CHQ scores were significantly higher in patients with unexplained chronic cough than in all other participants. Most importantly, no relationship was found between CHQ scores and capsaicin cough threshold. Furthermore, the CHQ more accurately discriminated between patients with chronic cough, patients with cough related to respiratory disorders, and normal subjects. The results suggest that the CHQ helps to identify patients with chronic cough unrelated to chronic respiratory diseases.

The usefulness of questionnaires, specifically the LCQ, is further confirmed by a retrospective study (**Kanemitsu, Y, Nimi, A, Matsumoto, H, Ito, I, Oguma, T, Inoue, H, Tajiri, T, Iwata, T, Nagasaki, T, Mishima M:** Cough assessed by the Leicester Cough Questionnaire (LCQ) in patients with cough variant asthma: impact of sex and gastric dysmotility) aimed at clarifying the relationship between cough-related quality of life (QOL) measures and variables derived from several clinical and functional assessments (lung function, exhaled nitric oxide, airway hyperresponsiveness assessments, sputum analysis and allergy tests). Symptoms related to gastro-oesophageal reflux and oesophageal dysmotility were also collected. It was found the LCQ scores weakly but significantly correlated with female gender and, most notably, with symptoms of gastric dysmotility. A relationship was also found with allergy related variables. On one hand, the results suggest that the LCQ outcomes are influenced by sex and dysmotility; on the other hand, they also confirm the relationship between airway disorders with those of the upper digestive tract [5].

The study by Xu et al. (**Xu, X, Chen, Q, Liang, S, Lv, H, Qiu, Z:** Prediction of gastroesophageal reflux-induced cough with questionnaire for patients with upper gastrointestinal symptoms) made use of another questionnaire, the so-called “GerdQ”, a patient-centred, self-assessment questionnaire to assist health care professionals in the diagnosis and effective managements of patients with gastro-oesophageal reflux disease (GORD) without initial specialist referral or endoscopy [6]. The purpose was to assess the predictive value of the GerdQ in the assessment of patients with reflux-related chronic cough who also underwent oesophageal impedance measurements. The diagnosis of GORD was also determined empirically by subsequent empirical antireflux therapy. The findings were that the sensitivity, positive and negative predictive values of GerdQ were comparable to those of variables derived from invasive and expensive procedures such as the intraluminal multi-channel impedance in the diagnosis of GOR in patients with acidic reflux. On the other, hand, the authors found less convincing relationships between GerdQ scores and coughing related to non-acidic reflux.

When two or more nerve impulses insist on a common neural substrate, the net resulting intensity of response will be lower than that expected from summation of the responses to the two stimuli acting separately. This physiological phenomenon has been termed “neural occlusion” [7]. Based on this, it can be questioned whether a respiratory sensation evoked experimentally alters the perception of another provoked sensation. This interesting and potentially important problem has been addressed by a study aimed at ascertaining if different respiratory sensations, namely dyspnoea and the urge to cough, interfere with the threshold and tolerance to a thermal pain stimulus applied on the skin of normal subjects (**Ebihara, S, Gui, P, Ebihara, T, Kashiwazaki, N, Ito, K, Kanezaki, M, Kohzuki, M**: Urge-to-cough and dyspnoea conceal perception of pain in healthy adults). It was found that both respiratory sensations “dose-dependently” increased threshold and tolerance to heat-induced somatic pain, indicating downregulation of pain perception by respiratory stresses. The findings also suggest a common central processing area for sensory inputs originating from somatically- and viscerally-innervated districts of the body. Whether painful sensation can modulate respiratory sensations remains to be ascertained.

Aspiration pneumonia associated with an impaired cough reflex has been implicated in several investigations performed on patients with respiratory and extra-respiratory diseases [8]. In keeping with this, a retrospective study performed on patients with recurrent pneumonia (**Ito, I, Ishida, T, Tachibana, H, Tomioka, H, Kadowaki, S, Tanabe, N, Niimi, A, Mishima, M:** Presence of dry cough symptom may prevent recurrent fever in patients treated for pneumonia) demonstrated a positive association between a weak cough reflex - as witnessed by the absence of cough during an episode of pneumonia - and the probability of developing recurrent pulmonary infections. Recurrence has been interpreted as a consequence of repeated aspiration of infectious material, most likely mucus, by the patients. Therefore, presence of cough during the early stages of pneumonia appears to predict a favourable outcome of the disease, thus confirming the protective role of cough during respiratory infections.

An interesting report from Slovakia and the UK adds to the growing evidence [9] of an important role played by nasal afferents in cough control (**Buday, T, Brozmanova, M, Biringerova, Z, Gavliakova, S, Poliacek, I, Calkovsky, V, Shetthalli, MV, Plevkova, J:** Urge to cough, cough sensitivity and intensity after nasal TRPM8 and TRPA1 agonists challenges). The main hypothesis was that cough could be modulated bidirectionally by appropriate stimulation of nasal afferents by means of two well-known compounds pertaining to the family of the TRP receptor agonists: the TRPA1, supposedly facilitating cough, and the TRPM8, possibly inhibiting it. In partial agreement with this possibility, it was found that following a nasal challenge with a TRPA1 agonist the urge to cough sensation to inhaled capsaicin was significantly enhanced, though actual cough was unchanged. Conversely, following a nasal challenge with TRPM8 agonists, both the urge to cough and the cough response, in terms of both sensitivity and intensity, to the same tussigenic agent were markedly reduced. Three interesting conclusions seem to emerge from this study. First, it confirms the bidirectional modulation of cough from nasal afferents; second, it discloses a potential mechanism for pharmacological cough control; third, it would appear that the urge to cough and the motor cough response are subjected, at least to some extent, to a different control mechanism.

### Treatment

Safe and effective therapies for acute and chronic cough remain a major area of unmet need [10]. Recently, a great deal of attention has been focused on the transient receptor potential (TRP) class of ion channels, that are expressed on airway sensory nerves and are believed to play a major role in regulating the afferent arm of the cough reflex [11]. Thus, the development of a clinically useful TRP antagonist, particularly of the TRPV1 subtype, has been the goal of many recent research programs. In a study by Smith et al. (**Smith, JA, Murdoch, R, Newlands, A, Khalid, S, Smart, K, Kelsall, A, Holt, K, Dockry, R, Woodcock, A**: The impact of a selective oral TRPV1 antagonist in patients with chronic cough), the effect of a potent, selective, oral TRPV1 antagonist, SB705498, was evaluated in 21 patients with unexplained chronic cough. End points measured were pharmacokinetic (PK) derived TRPV1 receptor occupancy, change in cough reflex sensitivity to inhaled capsaicin, objectively measured cough counts, and two subjective measures, Cough Quality of Life Questionnaire (CQLQ) and visual analog scale (VAS). Despite a 4-fold shift in capsaicin cough threshold, no difference was observed in objective cough counts or subjective end points compared with placebo. Despite a clear relationship between receptor occupancy and engagement of the TRPV1 receptor as evidenced by the shift in capsaicin cough threshold, no clinical efficacy parameter was improved, suggesting that TRPV1 receptor activation is not an important determinant of spontaneous cough frequency and that reductions in capsaicin cough reflex sensitivity do not necessarily predict antitussive effects in a population of patients with chronic unexplained cough.

In another study of patients with refractory, chronic cough, Ryan and colleagues evaluated 62 subjects treated with escalating doses of gabapentin vs. placebo in a 10-week therapeutic trial (**Ryan, NM, Birring, SS, Gibson, PG**: Gabapentin treatment for refractory chronic cough: a randomized controlled trial). Treatment with gabapentin significantly improved cough specific quality of life, the primary end point of the study, as well as cough frequency and cough severity. Cough reflex sensitivity to capsaicin was not affected. This study has since been published [12].

The two studies just discussed offer an interesting contrast, as the TRPV1 antagonist significantly diminished capsaicin cough sensitivity without demonstrating a clinically significant effect, whereas gabapentin achieved a significant clinical outcome without modulating cough reflex sensitivity to capsaicin.

Reminding us that not all therapy for cough need be pharmacologic, Chellini et al. described a novel surgical procedure successful in two subjects with refractory, chronic cough associated with excessive dynamic airway collapse (EDAC)(**Chellini, E, Gonfiotti, A, Magni, C, Innocenti, M, Jaus, M, Lavorini, F, De Francisci, A, Fontana, G, Macchiarini, P:** Membranous tracheobronchial reduction surgery for excessive dynamic airway collapse associated with intractable chronic cough). After membranous tracheobronchial reduction surgery, patients had demonstrable improvement/resolution of the inward bulging of the posterior membrane that previously had occurred during expiration and voluntary coughing, with a concomitant improvement in lung function and marked reduction in spontaneous cough.

Ogawa and colleagues reported further work from their group on the topic of chronic cough associated with sensitization to the fungus *B. adusta* (**Ogawa, H, Fujimura, M, Takeuchi, Y, Makimura, K:** The significance of sensitization to *Bjerkandera adusta* in the successful treatment of chronic idiopathic cough patients). The investigators observed that, in 10 subjects with chronic idiopathic cough, a 14-day course of low-dose (50 mg/day) itraconazole therapy was more likely to be beneficial in subjects with a positive immediate cutaneous reaction to *B. adusta*.

## Conclusions

The 26 presented papers were all notable for their high quality. Studies presented in these posters have provided new information that should improve our knowledge on the basic physiology and pharmacology of cough, and the peripheral and central neural mechanisms involved in the generation of the cough motor pattern. In addition, in the clinical science section, studies reporting potential new antitussive agents and further characterisation of cough symptoms and perception have provided a base for the fruitful strategies for the development of novel antitussive therapies and cough management. The extensive discussion captured the attention of the auditorium and stimulated an in-depth exchange of ideas on some of the most urgent issues in basic and clinical cough research.

## Competing interests

The authors declare that they have no competing interests.

## Authors’ contributions

All four authors participated and contributed equally to the preparation of this article. All authors have read and approved the final manuscript.
